# 

**Published:** 1894-11

**Authors:** 


### Southern Medical Record.

A Monthly Journal of Medicine and Surgery.

VOL. XXIV. ATLANTA, GA., NOVEMBER, 1894. No. 11.

EDITORS:

A. W. GRIGGS, M. D. WILLIS F. WESTMORELAND, M. D.
J. McFADDEN GASTON, M. D. LOUIS H. JONES, M. D.
DAN. H. HOWELL, M. D., BUSINESS MANAGER.

Entered at the Postoffice at Atlanta, Ga., as Second-Class Matter.

Foote & Davies Co., Atlanta, Ga.

